# Parliament and the Professions

**Published:** 1918-11-30

**Authors:** 


### PARLIAMENT AND THE PROFESSIONS.

### Physical Training Under the Education Act.

THE President of the Board of Education informed Colonel Yate that there are now on the Board's staff seven expert inspectors of physical training, and it will probably be necessary to increase this number in the near future. Acting on the Board's suggestion, many local education authorities are now appointing organisers of physical exercises; fifty-nine such appointments have already been made. In considering schemes submitted by local education authorities under the Education Act, 1918, the Board will have regard to the importance of securing satisfactory provision for physical training. Physical inspectors are to be appointed in England.

### Strikes of Asylum Officers.

MR. T. WILSON asked the Home Secretary for particulars of recent strikes on the part of asylum attendants. Mr. Brace said that there have been a few recent cases of actual or threatened strikes at asylums. The Board of Control—in which the Lunacy Commissioners are now absorbed—have no power to regulate rates of pay or conditions of service of asylum officers; these are matters for the local authorities by whom the asylums are established and managed. The provision of means for the settlement of questions relating to the conditions of employment is engaging the attention of the Ministry of Labour.

### The Red Cross Society.

MR. SNOWDEN asked the Financial Secretary to the War Office whether the Red Cross Society is in any way under the control of the War Office in regard to compensation for its servants who may be killed in action or who contract diseases and become incapacitated on Red Cross service; and if the Red Cross are under any legal obligation to give pensions or compensation in such cases. Mr. Forster replied that the War Office has no control of the funds placed by the public in the hands of the Red Cross organisation. But where Red Cross *personnel* is employed with War Office approval on military duties with British Forces the British Government gives pension or compensation as for military *personnel*.

